# Epidemiology, genetic epidemiology and Mendelian randomisation: more need than ever to attend to detail

**DOI:** 10.1007/s00439-019-02027-3

**Published:** 2019-05-27

**Authors:** Nuala A. Sheehan, Vanessa Didelez

**Affiliations:** 1 Department of Health Sciences, University of Leicester, Leicester, UK; 2 Leibniz Institute for Prevention Research and Epidemiology-BIPS, Bremen, Germany; 3 Faculty of Mathematics and Computer Science, University of Bremen, Bremen, Germany

## Abstract

In the current era, with increasing availability of results from genetic association studies, finding genetic instruments for inferring causality in observational epidemiology has become apparently simple. Mendelian randomisation (MR) analyses are hence growing in popularity and, in particular, methods that can incorporate multiple instruments are being rapidly developed for these applications. Such analyses have enormous potential, but they all rely on strong, different, and inherently untestable assumptions. These have to be clearly stated and carefully justified for every application in order to avoid conclusions that cannot be replicated. In this article, we review the instrumental variable assumptions and discuss the popular linear additive structural model. We advocate the use of tests for the null hypothesis of ‘no causal effect’ and calculation of the bounds for a causal effect, whenever possible, as these do not rely on parametric modelling assumptions. We clarify the difference between a randomised trial and an MR study and we comment on the importance of validating instruments, especially when considering them for joint use in an analysis. We urge researchers to stand by their convictions, if satisfied that the relevant assumptions hold, and to interpret their results causally since that is the only reason for performing an MR analysis in the first place.

## Introduction

In many areas of application, it is important to be able to distinguish a causal association from a non-causal one to assess the relationship between a treatment, or exposure, *X*,  and an outcome *Y*. In econometrics research, for example, interest might focus on whether programmes for the unemployed are actually effective in increasing the chances of returning to work, or whether more years of schooling increase the expected salary from future jobs. In epidemiological research, establishing the causal effect of a treatment or a modifiable exposure on a health outcome is crucial for informing decisions about treatment delivery and public health interventions. The randomised controlled trial (RCT) is the accepted ‘gold standard’ for determining causality, since randomisation to the exposure renders all other explanations for an observed association unlikely. When it is difficult, or impossible, to randomise *X* and unobserved confounding cannot be ruled out, an established approach in econometrics is to switch, if possible, to the next best thing: find a variable that, is closely related to *X*, does not directly affect *Y*, and can either be actively randomised by the investigator or is randomised by nature. Such a variable is called an instrumental variable (IV) (Angrist and Pischke 2009).

As an example of an actively randomised IV, we can conceive of a trial where unemployed individuals are randomly allocated to either participate in or abstain from a certain programme and their employment status recorded a year later, or where individuals are randomly assigned to a particular treatment and their health status monitored at a later time point. As not all participants comply with their allocation—some refuse and others enter the programme or take the treatment even if assigned to the control group—the actual exposure may differ from that dictated by the randomisation. Since the actual behaviour is affected by external circumstances or an individual’s attitudes and preferences, the exposure–outcome association is typically confounded. However, due to the fact that the allocation was properly randomised, it can be exploited as an IV which, in this case, is a randomised incentive or encouragement providing an imperfect way to assign *X*.

For the schooling example where randomisation would not be possible, a solution is to use an individual’s month of birth as an IV as this is related to years of schooling via the cutoff date for enrolling students in many educational systems. Here, nature (through the birth month), rather than the investigator, provides the randomisation. In epidemiology, as a direct result of the recent explosion in findings from genetic association studies, there has been a heightened interest in using genetic variants related to exposures of interest as IVs. Again, these are IVs where nature randomises between parents and offspring according to Mendel’s laws of inheritance, and the term Mendelian randomisation (MR) has now become standard for instrumental variable methods that use genetic IVs (Davey Smith and Ebrahim 2003; Didelez and Sheehan 2007b; Lawlor et al. 2008a). In short, IVs essentially constitute an imperfect way of randomising the actual quantity *X* of interest. As it is imperfect, any conclusions drawn using an IV are weaker than those from a randomised controlled trial with full compliance. However, provided the underlying assumptions are satisfied, it does permit consistent inference about the causal effect of *X* on *Y* despite unobserved confounding (Greenland 2000).

In this article, we outline the basic concepts, benefits and challenges of IV analyses for epidemiological applications. We will focus particularly on the use of genetic IVs and hence on Mendelian randomisation studies, noting where these differ from other applications. To see how IVs enable causal conclusions, we begin by formalising the difference between association and causation and introduce causal effect measures. We define and illustrate the notion of an IV and emphasise the importance of establishing the validity of a candidate IV. To illustrate the issues for drawing causal conclusions, we consider the main statistical approaches using a single IV in a ‘one-sample’ setting where individual-level data are available on all observable quantities. We then briefly discuss the additional issues and complications that arise with multiple IVs and also in ‘two-sample’ settings where the IV–exposure and IV–outcome associations are derived from separate studies. We conclude with a discussion of current developments and challenges for Mendelian randomisation. We will use directed acyclic graphs (DAGs) throughout to illustrate the conditional independencies implied by the joint distribution of a set of variables (Dawid 1979; Pearl 2000).

## Basic causal concepts

To fully disentangle causal from associational concepts, we want to formally distinguish between the two. This is an important distinction in observational epidemiology, for example, as an exposure might be associated with a disease outcome but an intervention that changes the exposure levels will not necessarily affect disease risk and so could be ineffective unless the association is causal. Specifically, we say that a variable *X* is associated with another variable *Y* if the observation of one is informative, or predictive, for the other. Such association is encapsulated by the usual conditional probability notation, whereby $P(Y=y \mid X=x)$ describes the distribution of *Y* given that we observe $X=x$ has occurred. We argue, as others have done, that the notion of intervening in a particular system is fundamental to any formal approach to causality even though this is not always explicit (Pearl 2000; Hernán 2004; Didelez and Sheehan 2007a). Thus, when we say that *X* causes *Y*, we mean that an intervention on *X* that sets it to a given value is informative for *Y*. The problem of causal inference we consider here is that of obtaining information on what might happen under intervention from observational data where the desired intervention had not actually taken place.

### Formal framework for causality

We adopt the notation $\mathrm{do}(X=x)$, as suggested by Pearl (2000), to represent the intervention of setting *X* to a value *x* as opposed to allowing *X* to assume this value naturally. That “association is not causation” is reflected in the fact that the two conditional distributions $P(Y=y \mid \mathrm{do}(X=x))$ and $P(Y=y|X=x)$ are not necessarily the same. The former depends on the value *x* only if *X* is causal for *Y*. It corresponds directly to what we would observe in a randomised study (with perfect compliance). The latter depends on the value *x* for other reasons besides causality, such as when there is confounding or reverse causation of the *X*–*Y* relationship, and corresponds to the distribution we obtain from an observational study. To illustrate the difference, consider a hypothetical situation with binary variable *X* indicating whether an individual’s fingers are stained or not, and a binary outcome *Y* indicating the presence or absence of lung cancer. Then, $P(Y=y \mid X=x)$ describes how lung cancer risk can be predicted from inspection of someone’s fingers because they are informative for smoking which is, in turn, informative for lung cancer. However, an intervention on *X*, such as removing the finger staining, would render this no longer informative for lung cancer risk and so we would expect that $P(Y=y \mid \mathrm{do}(X=x))$ would not depend on *x* (Sheehan et al. 2011).

Other formal frameworks exist, the most prominent of which is the potential outcomes approach, where *Y*(*x*) denotes the value of the outcome *Y* if *X* were set (by a well-defined intervention) to *x* (Hernán 2004). In the case of a binary exposure, we have two potential outcomes *Y*(1) and *Y*(0), only one of which can ever be observed making the other one counterfactual. For our purposes, we can regard the two concepts as equivalent, i.e. $P(Y=y \mid do(X=x))=P(Y(x)=y)$ (Didelez and Sheehan 2007b).

### Causal effects

We define a causal effect of *X* on *Y* to be some measure of how *Y*, or its distribution, behaves under different interventional settings of *X*. A popular causal parameter is the average causal effect (ACE) describing the average change in *Y* from setting *X* to some value $x_2$ compared with another (e.g. baseline) value $x_1$. It is defined as

$$\begin{aligned} \mathrm{ACE}(x_1,x_2 )=E(Y\mid \mathrm{do}(X=x_2 ) )-E(Y\mid \mathrm{do}(X=x_1 ) ). \end{aligned}$$

For binary outcomes, the above is the risk difference for the two possible settings of *X*. The causal relative risk (CRR), defined as

$$\begin{aligned} \mathrm{CRR}=\frac{P(Y=1 \mid \mathrm{do}(X=x_2))}{(P(Y=1\mid \mathrm{do}(X=x_1 ))}, \end{aligned}$$

and the causal odds ratio (COR), defined analogously, are the more common parameters for a binary outcome *Y*. Observational data only permit inference on causal parameters if suitable conditions are satisfied; we then say that the causal parameter is identifiable. One such condition is that all (or a sufficient set of) confounders have been appropriately taken into account, e.g. by standarisation or using inverse probability weighting (Hernán and Robins 2006b). When this assumption is not reasonable and unobserved confounding is suspected, instrumental variable methods can provide an alternative approach.

The above ACE, CRR and COR are defined in terms of changes across the whole population and are therefore population parameters. In some situations, it may be more relevant to look at causal effects within (possibly latent) subgroups, especially when there is *effect modification* whereby individuals in different subgroups respond differently to exposure.

## Instrumental variables

We have motivated IVs intuitively via imperfect randomisation by the investigator or nature. Now we address the formal conditions that make an IV a valid tool for drawing causal conclusions. We will use the notation $A \bot \!\!\!\bot B \mid C$ to express that *A* is conditionally independent of *B* given *C* (Dawid 1979).

### Core IV conditions

We denote the exposure by *X*, the outcome by *Y* and the unobserved confounding between *X* and *Y* by *U*. So *U* is a set of variables that could be sufficient to adjust for confounding of the *X*–*Y* association if they could be measured. Then, a third observable variable *G* is an instrumental variable (IV), or an instrument for the causal effect of *X* on *Y*, if:

1. $G \bot \!\!\!\bot U$: *G* is (marginally) independent of *U*,  i.e. the instrument is not associated with the unobserved confounding between *X* and *Y*;
2. $G \bot \!\!\!\bot \!\!\!\!/\;X$: *G* is associated with the exposure *X*;
3. $G \bot \!\!\!\bot Y \mid (X,U)$: *G* is conditionally independent of *Y* given the exposure *X* and confounding *U*, i.e. *G* and *Y* would not be associated after adjusting for both *X* and *U*.

**Fig. 1:**
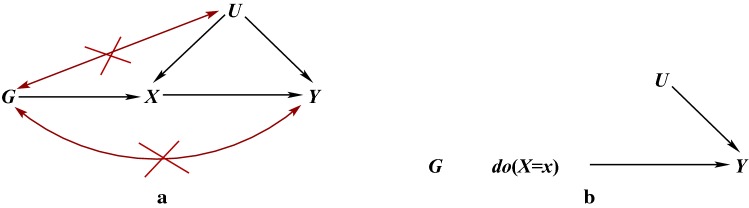
DAGs representing, **a** the core conditions for *G* to be an IV for the association between *X* and *Y* in the presence of unobserved confounding factors *U*,  where the red lines represent open paths that are not allowed by these conditions and, **b** the effect of an intervention on *X* on the joint distribution of *G*, *X*, *Y*, *U* under the structural assumption. A bi-directed edge represents an association that is possibly via a common graph ancestor

We refer to the above as the IV core conditions and they are uniquely encoded in the directed acyclic graph (DAG) in Fig. 1a (Greenland 2000; Didelez and Sheehan 2007b; Didelez et al. 2010). The first condition is represented by the absence of an edge between *G* and *U* and all other paths in the graph between *G* and *U* are blocked by a collider. The second condition is represented by the edge between *G* and *X*. However, it should be noted than many IV methods actually require this association to be linear, e.g. $\mathrm{Corr}(G,X) \ne 0$. For the third condition, note that as *X* is a collider on the $G \rightarrow X \rightarrow Y$ path, conditioning on *X* alone opens another path between *G* and *U*. Conditioning on both *X* and *U* hence blocks all paths and there is no other edge between *G* and *Y*. Equivalently, the joint distribution of the four variables factorises in the following way:

$$\begin{aligned} p(y,x,u,g)=p(y \mid x,u)p(x \mid u,g)p(u)p(g). \end{aligned}$$

We will often assume that this joint probability distribution is faithful to the graph in Fig. 1a by which we mean that every conditional independence in the probability distribution corresponds to a separation in the graph and so every edge corresponds to an association (Spirtes et al. 2000).

The three core conditions above describe how the four variables are related ‘naturally’. We require a fourth assumption to infer anything about the causal effect of *X* on *Y*. In particular, we should be able to envisage intervening on *X* without modifying the distributions $p(y \mid x,u), p(g)$ and *p*(*u*),  i.e. these should not change under $\mathrm{do}(X=x)$. In other words, the distributions of *G* and *U* and the conditional distribution of *Y*, given *X* and *U*, are of the same form regardless of whether *X* has arisen “naturally” or has been set by external intervention. We refer to this as a structural assumption: it is required to link the observational and the interventional regimes and we say that the graph is causal with respect to *X* (Lauritzen 1996). Mathematically, the joint distribution under such an intervention is given by

4. 1 $$\begin{aligned} p(y,x,u,g \mid \mathrm{do}(X=x^* )=p(y \mid x^*,u)I{(x=x^*)}p(u)p(g), \end{aligned}$$

where $I{(x=x^*)}$ is the indicator function taking the value 1 if $x=x^*$ and 0 otherwise. This structural assumption is often not stated explicitly, but implied by the specific structural model used for the analysis, e.g. a linear structural equation model. We state it explicitly as it is also relevant to non-parametric inference, e.g. when testing or computing bounds (see below).

The structural assumption essentially defines the class of (possibly hypothetical) interventions for which the IV can be used. In an MR study, for example, where *X* represents an individual’s BMI and it is suspected that *U* includes diet and amount of exercise, then use of a genetic variant in the FTO gene as an IV to estimate the causal effect of BMI on some health outcome (Frayling et al. 2007; Timpson et al. 2009) is only informative for potential interventions that change BMI but not diet and amount of exercise. This is quite different from actual RCTs that target BMI where interventions often do consist of changes to diet or exercise. In other examples, such as exposure to alcohol or smoking, an intervention that changes the law so that people are prevented from smoking or drinking could mean that they adapt their lifestyle in other ways to compensate and hence the required structural assumption might not be satisfied. It should be noted that in many applied MR studies, the structural assumption (1) is implicitly assumed when testing for and estimating causal effects without any discussion of its appropriateness for the intervention under consideration. This is a critical omission.

Graphically (see Fig. 1b), an intervention on *X* removes all the directed edges into *X* and renders *G* marginally independent of *Y* and *U*. This independence is related to, but should not be confused with, the exclusion restriction (Hernán and Robins 2006a) typically found instead of core conditions 1 and 3 in IV contexts where the IV is controlled by the investigator (see below).

### Two types of instrumental variables

As noted above and in the initial motivation, there are two general types of IV: those that are under the control of, and randomised by, the investigator but with imperfect compliance, and those that are not, but are instead in some sense ‘randomised’ by nature. Much of the literature on IVs assumes the first case, whereas some applications, in particular Mendelian randomisation, fall into the second category. As the two are not always clearly distinguished in the MR literature (Thanassoulis and O’Donnell 2009; Burgess and Thompson 2013, 2015; Howell et al. 2018), we feel it is important to highlight and discuss the differences here.

In well-conducted RCTs, with valid randomisation in a double-blind or comparative setting but with imperfect compliance, it is a fact, and thus is not required as an assumption, that the IV is not affected by any baseline variables or factors. This is because randomisation breaks any association with measured or unmeasured pre-randomisation characteristics predictive of the outcome *Y*. Hence, the IV will not be affected by any parts of *U* that are prior to randomisation. If the IV were randomised by the investigator, the only way in which core conditions 1 and 3 could be violated is by a ‘direct’ effect of the IV either on *Y* or on any post-randomisation parts of *U*,  i.e. an effect on *Y* that is not mediated via the exposure of interest *X*. This is the exclusion restriction and, in the potential outcomes framework, is expressed as $Y(g,x)=Y(x)$, i.e. *G* has no direct causal effect on *Y* via a route other than through *X*. Hence, the exclusion restriction can replace core conditions 1 and 3 in these situations. In contrast, for IVs of the second type where the instrument is not controlled as in Mendelian randomisation applications, we have to justify not only that the instrument has no effect on the outcome other than through *X*, but that it is also not affected by *U* and not otherwise confounded with *Y*.

Distinguishing between the two types of IV is also relevant with respect to the causal parameter being targeted. In partial compliance situations, it is common to estimate the average causal effect among ‘compliers’ only, i.e. those individuals who would comply with the assignment whatever their assigned group. The target parameter is then called the ‘local average treatment effect’ (LATE) or complier causal effect. The LATE is identified using only the exclusion restriction and a monotonicity assumption, the latter stating that ‘defiers’ do not exist when *G* and *X* are both dichotomous (Imbens and Angrist 1994; Greenland 2000) For an intention-to-treat (ITT) analysis under partial compliance, an observed association between the randomised allocation and outcome can only be due to the treatment having a causal effect on the outcome since the group assignment itself cannot affect the outcome. Under the exclusion restriction and monotonicity assumptions, the association has a causal interpretation as the effect of treatment assignment for a population with comparable compliance behaviour. In placebo controlled trials, this would be a conservative estimate of the actual complier causal effect, but not necessarily in other types of trial.

An argument in favour of targeting this local parameter is that in real life, we often cannot enforce *X* to be a particular value *x*, and since we can only provide incentives, the only effect that is of relevance is the effect on those who comply. Arguments against it are based on the fact that the ‘compliers’ are an unidentifiable latent subgroup and that as incentives outside a trial may not be comparable to the incentive used in the trial, a population parameter is the relevant quantity to target. Importantly, when the instrument is not an incentive controlled by the investigator, we would argue that the term ‘complier’ makes little sense. In an MR context, for instance, it would refer to the subgroup of individuals whose phenotype would always correspond to their genotype whatever genotype was assigned by nature. For a detailed discussion of issues around the two types of IVs, see Joffe (2011) and Dawid and Didelez (2012). Other authors have commented on the inappropriateness of this parameter for MR analyses since the degree of adherence to a non-explicit trial protocol cannot be determined. There are also issues with interpreting this parameter if the IV is not causal, as compliance must then be defined with respect to a latent causal factor associated with the IV. (Swanson et al. 2017; Swanson and Hernán 2018).

### Establishing validity for a candidate IV

Finding an IV can be a challenge. One problem is that the validity of core conditions 1 and 3 cannot be easily checked empirically as they involve the unobservable *U*. Instead, we need to use subject matter knowledge, indirect empirical evidence or additional assumptions to justify them which, in turn, require a deep understanding of the issues involved. In contrast, core condition 2 can (and should) be easily tested by investigating the *G*–*X* association. For reasons that will become clearer later, we say that the IV is strong if this association is ‘large’ and weak otherwise. It is sometimes wrongly suggested that the validity of core conditions 1 and 3 can be verified by checking that *Y* is not associated with *G* given *X* alone (possibly due to confusion with the exclusion restriction) or that *Y* and *G* are marginally independent—but these independencies are neither implied nor required for the core conditions to hold. As noted in “Core IV conditions”, *X* alone does not block all paths between *G* and *Y* in Fig. 1a since *X* is a collider and, trivially, these paths are not blocked by the empty set as there is a directed path linking *G* to *Y*. In the special case of all variables being binary (or discrete with few levels), conditions 1 and 3 impose restrictions on the observable variables in the form of inequalities which can be used for detecting gross violations of these core conditions (Balke and Pearl 1994; Bonet 2001) (see “Bounds on causal effects” below).

Despite the difficulty, the core conditions should be evaluated more systematically than is typically done in the literature. MR studies, unlike other areas of application, have the potential advantage of good background biological information with which to justify these for a genetic IV. Firstly, core condition 1 means that the genetic variant must not be associated with the sort of confounding you might expect for the particular *X*–*Y* relationship considered. When some confounders are in fact measured, it is quite common in practice to check association with these and interpret no observed association as support for core condition 1 under the strong (and also untestable) assumption that any unobserved confounding would behave in a similar way (Davey Smith et al. 2007; Lawlor et al. 2008b; Palmer et al. 2012; Au Yeung et al. 2013; Burgess et al. 2017a). It is also argued that because genes are randomly assigned (conditionally on parental genes) at meiosis, they should be reasonably immune to confounding of the *X*–*Y* association across the population (Lawlor et al. 2008a). However, one has to be careful that the particular variants under consideration are not also associated with lifestyle factors that could, in principle, confound the association between *X* and *Y*. A more comprehensive understanding of the underlying biological pathways is required to justify core condition 3 since all other pathways between the gene and outcome must be ruled out. If some sensible assumption can be made about the direction of the unobserved confounding, simple tests comparing the IV estimate with the ordinary observational estimate can also be informative (Glymour et al. 2012). Generally poor reporting of MR studies has been commented on elsewhere in the literature and several suggestions have been made for improvement (Davies et al. 2015; Glymour et al. 2012; Swanson and Hernán 2013; VanderWeele et al. 2014; von Hinke et al. 2016).

**Fig. 2:**
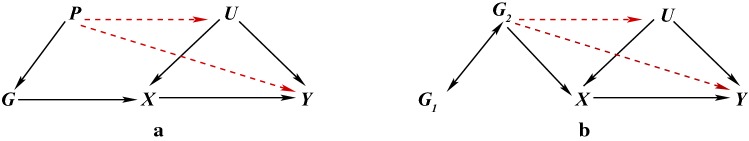
DAGs illustrating possible violations of the core IV assumptions due to population stratification (**a**), or linkage disequilibrium (**b**), where dashed red edges create violations. A bi-directed edge represents an association, possibly via a common graph ancestor

Violations of the core IV conditions can occur for several reasons. These have implications for causal inference and analyses can be sensitive to such violations (Hernán and Robins 2006a; Didelez et al. 2010). In MR studies, a plausible violation arises from population stratification, where different sub-populations of the study have different allele frequencies and also happen to have different distributions of unobserved risk factors for disease or different disease prevalences due, for example, to different cultural lifestyles. The former could yield an association between *G* and *U*,  while the latter induces an association between *G* and *Y* other than via *X* and *U* as depicted in Fig. 2a. If population stratification is fully understood, this violation can be handled by study design, e.g. by carrying out the analysis separately in each sub-population as is standard in epidemiological studies.

Core condition 2 does not state that the IV has to be causal for *X*,  although this is naturally the case when the IV is a properly randomised incentive and causality of this relation is actually a requirement for some IV methods in that particular situation (Swanson and Hernán 2018). Note also that the potential outcomes framework, as assumed in von Hinke et al. (2016) for instance, explicitly assumes that the IV is causal. Thus, using a genetic IV, $G_1,$ that is not actually causal for the exposure of interest and is only associated because it is in linkage disequilibrium with an unobserved variant, $G_2$, which *is* causal for *X*,  is not a violation. However, it would be an issue if the unobserved variant, $G_2$, were also associated with the outcome, *Y*, via a route other than through *X* (see Fig. 2b). In particular, core condition 1 for $G_1$ would be violated if $G_2$ were associated with *Y* via the confounders *U*, whereas core condition 3 would not hold if the unobserved variant were associated with *Y* via another mechanism that does not involve *X*.

**Fig. 3:**
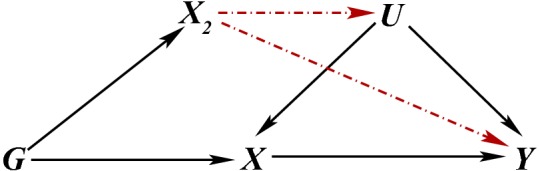
DAG illustrating violation of core condition 3 (dashed red lines) for a candidate IV *G* via its pleiotropic effects on *Y* via another exposure $X_2$

Furthermore, genetic variants proposed as candidate IVs from genetic association studies are likely to have pleiotropic effects on other exposures besides *X*, thus potentially violating core condition 3 if these are unmeasured and cannot be adjusted for (Fig. 3). In the case where measurements on the pleiotropic variants are available, constrained instrumental variable methods can be used to find optimal instruments for the exposure of interest and for appropriate adjustment of causal analyses (Jiang et al. 2019). Directed acyclic graphs are useful to represent what is believed about the underlying biology and to check the core assumptions (Didelez and Sheehan 2007b).

The exploitation of well-studied genetic variants with known functionality is essential for the success of MR studies. In particular, although current genome-wide association studies are discovering more and more associations between single nucleotide polymorphism (SNP) variants and epidemiological exposures, the gene–exposure associations are often weak and may not even be reproducible making them unsuitable IV candidates (Taylor et al. 2014). More to the point, many of these variants are not yet sufficiently well understood to validate as IVs and researchers need to be prepared to continually review their suitability as IVs as the functional knowledge becomes available and through incorporation of any other relevant external knowledge (Tchetgen Tchetgen et al. 2013). Importantly, if there is insufficient prior knowledge about the genetic or confounding mechanisms to justify the core conditions, it is possible that results from an IV analysis indicating a causal effect may very well have an alternative non-causal explanation.

## Principles of inference with IVs

We now explain the principles underlying statistical inference about causal effects using an IV. We will not focus on the details here as these depend on the specific setting, e.g. continuous or binary outcome, and are discussed elsewhere. We begin by asking if there is a causal effect of *X* on *Y* at all. We then consider whether lower and upper bounds can be derived for this causal effect. Finally, we address the issue of getting a point estimate of the causal effect. Answering these questions in turn requires increasingly more restrictive assumptions.

### Testing for a causal effect by testing for a *G*–*Y* association

When our interest is purely in confirming whether there is an average causal effect of *X* on *Y* in the first place, data on a valid IV *G* (satisfying the core conditions) and the outcome *Y*, together with the structural assumption (1) and faithfulness, are sufficient, i.e. we do not require data on *X*. More specifically, as reasoned below, we just need to test for a (marginal) association between *G* and *Y*. This has some analogies with the intention-to-treat (ITT) analysis under partial compliance discussed earlier, although the two should not be confused.

**Fig. 4:**
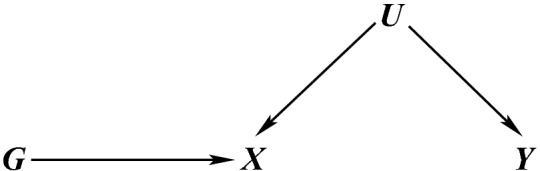
DAG illustrating the null hypothesis of no causal effect of *X* on *Y* by the absence of an edge between *X* and *Y* rendering *G* and *Y* marginally independent

We will define the ‘causal null hypothesis’ of interest to mean the absence of an $X \rightarrow Y$ edge as depicted by the DAG in Fig. 4. We note that this is ignoring the possibility of causal effects in subgroups defined by *U* which cancel each other out. Under the core conditions, any marginal association between the IV, *G*, and the outcome, *Y*, can only occur if *X* has a causal effect on Y because there is no other pathway between *G* and *Y* that creates an association (Fig. 1a). As shown in Fig. 4, when there is no causal effect of *X* on *Y*, *G* and *Y* are marginally independent, i.e. there is no unblocked path between them since the path $G \rightarrow X \leftarrow U \rightarrow Y$ is blocked in the absence of conditioning on the collider *X*. The reverse reasoning is trickier. Lack of evidence for a *G*–*Y* association can mean several things: there is no causal effect, or the power is too low, or the IV is too weak. In rare cases, it could also happen that, due to interactions with unobserved factors, positive and negative subgroup effects ‘cancel’ each other out so that no overall effect is detectable. These issues are discussed in more technical detail in Didelez and Sheehan (2007b).

Any suitable statistical test for association can be used in this context, e.g. a Chi-squared test if *G* has three levels and *Y* is binary. Typically, a regression of *Y* on *G* (which can also accommodate possible observed covariates) is used and a statistically significant association is evidence for the presence of a causal effect of *X* on *Y*. Importantly for MR applications and in contrast with the ITT estimate in a partial compliance type setting, we note that the estimate of the *G*–*Y* association is generally not interpretable in terms of a causal effect. Neither does it permit inference about the magnitude nor sign of a causal effect (Didelez and Sheehan 2007b; Burgess and Small 2016; Swanson et al. 2018). It is purely a test for a causal effect and further assumptions must be made to obtain a point estimate of such an effect should it seem likely to be present. Hence, it is good practice to start an IV analysis by establishing what conclusions can be drawn from the *G*–*Y* association alone without additional assumptions. All methods that yield an estimate, standard error and confidence interval for the causal effect of *X* on *Y* make further parametric (or semi-parametric) assumptions over and above the core conditions. For instance, it may happen that the *G*–*Y* association is not statistically significant, but subsequent estimation of the causal effect of *X* on *Y* yields a significant result. We should then bear in mind that the apparent extra information ‘gained’ is mainly due to the additional modelling assumptions that were made. As these implicitly or explicitly involve the unobservable factors subsumed in *U*, they are empirically untestable. Furthermore, it is common to assume that *X* is measured without error. All these additional assumptions are themselves new sources of bias if violated, and resulting estimates and standard errors can be regarded as less reliable.

Finally, we point out that the test for a *G*–*Y* association to assess the presence of a causal effect is also valid in case-control studies without any further adjustment or additional assumptions other than a valid IV (Didelez and Sheehan 2007b). So, even if sampling is conditional on the outcome *Y*, we still expect a *G*–*Y* association only if *X* has a causal effect on *Y*. This is important because IV estimation in a case-control study is not straightforward, whereas a test for the *G*–*Y* association is very simple (Didelez et al. 2010; Bowden and Vansteelandt 2011). For example, in an investigation into the possible causal effect of homocysteine level on stroke risk, the odds ratio for the genotype-stroke (*G*–*Y*) association, using a dichotomisation of the MTHFR C677T polymorphism into TT and CC carriers as a genetic IV, was found to be significant at 1.26 with 95 % CI (1.14, 1.40) (Casas et al. 2005). If the MTHFR gene is a valid instrument for the effect of homocysteine on stroke, this result provides evidence for the presence of such a causal effect.

### Bounds on causal effects

In some cases, it is possible to obtain some quantitative information about the size of the causal effect in the form of lower and upper bounds using only the core IV conditions without further parametric assumptions. This is possible when *G*, *X*, and *Y* are discrete with few levels and data on all three variables are available from a single sample. In an MR study, for example, we might have a genetic IV with three levels, a binary exposure and a binary outcome. It is important to note that the bounds are not confidence intervals for the causal effect. The interpretation of the bounds is that the data are compatible with values of a causal effect anywhere between the lower and upper bound. We do not go into technical details here as these are provided elsewhere (Manski 1990; Balke and Pearl 1994; Palmer et al. 2011a).

Returning to the example above (“Testing for a causal effect by testing for a *G*–*Y* association”), we consider bounding the causal effect of dichotomised homocysteine level (low/high) on presence or absence of cardiovascular disease (CVD) using the MTHFR genotype (now with all three levels) as an IV (Palmer et al. 2011a). Since the data come from a case–control study, the analysis is performed by converting back to the required population frequencies under plausible assumptions about the prevalence of CVD (Didelez and Sheehan 2007b). With a prevalence of 6.5%, the ACE (causal risk difference) lies between $-\,0.0895$ and 0.7344 while assuming a prevalence of 2%, the bounds are slightly wider and the ACE lies between $-\,0.065$ and 0.7644. Alternatively, the bounds can be given for the CRR (causal relative risk) and are 0.1272 and 41.5740, respectively, in the latter case. While we previously reported the IV–outcome association for this example as supporting the presence of a causal effect of homocysteine on stroke risk, the bounds computed here are all too wide to confirm this as they all include the null hypothesis of ‘no effect’. This may be partly due to the fact that the test in Casas et al. (2005) was based on a meta-analysis while the bounds above were calculated using only a subset of the data which was less informative.

The fact that we can bound the causal effect is interesting in two regards. Firstly, it illustrates that even though the core IV assumptions do not imply any (conditional) independencies among the observable variables (*G*, *X*, *Y*) they still impose some restrictions leading to such bounds, and these restrictions can be exploited to test the validity of the core IV conditions to a certain extent. Secondly, the bounds are ‘tight’, meaning that nothing more precise can be said about the causal effect without further assumptions which underlines the necessity of the latter if an effect estimate is desired—especially if the calculated bounds are too wide to be informative (Balke and Pearl 1994). Thus, for the above example, any derived estimate of the effect of homoscysteine level on stroke risk will depend on the additional assumptions that are made for point estimation.

**Fig. 5:**
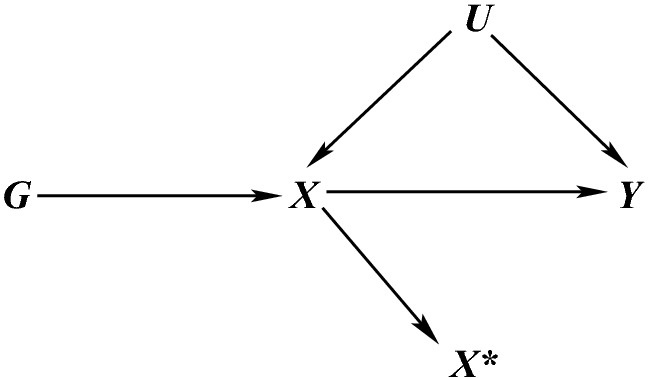
DAG illustrating the case where $X^*$ is an imperfect measurement of *X* (e.g. after dichotomising) and where *G* is not a valid IV for the causal effect of $X^*$ on *Y* since $G \bot \!\!\!\bot \!\!\!\!/\;Y \mid (X^*,U)$

Note that a major limitation is that if *X* is continuous, no bounds or other restrictions can be derived from the core IV assumptions, i.e. there are no testable implications of the IV assumptions and a parametric approach is thus required for causal inference (Bonet 2001). Conclusions drawn for a continuous exposure are hence completely reliant on the relevant parametric modelling assumptions. This is especially limiting in MR analyses where the exposure is typically continuous and is arguably the reason why few examples of computing the bounds can be found in the literature. In fact, when the exposure of interest is continuous, it may be unwise to dichotomise the exposure as the chosen IV might not be valid for the dichotomised variable as illustrated in Fig. 5 (Didelez and Sheehan 2007b; Glymour et al. 2012; VanderWeele et al. 2014; Swanson 2017). When the bounds can be computed, they tend in practice to be wide and often include the null as in the above example and are so deemed ‘uninformative’. We note that this is not a poor property of the method, but is rather a property of the data: MR data are often ‘uninformative’ in this sense due to the weak IVs that are typically used and possibly only small true causal effects, if any (see “IV estimation in linear and additive structural models” below). The width of the bounds depends on the strength of the IV and the amount of confounding. However, in contrast with Burgess et al. (2017b), we recommend that they be computed in the case where variables are genuinely discrete, if only to assess how much can be inferred without further assumptions (Richardson et al. 2014). In particular, bounds that do *not* include the null causal effect could lend considerable weight to reported causal findings. They can be calculated easily in Stata (Palmer et al. 2011a) and using either of two R packages, bpbounds and ivtools, that have recently been made available on CRAN. When several IVs are available, bounds can be calculated for each IV separately and the intersection of all such bounds considered. Likewise, bounds can be calculated for a combined IV (Swanson 2017).

### Estimation with instrumental variables

As already noted, estimation of the causal effect requires additional modelling assumptions over and above the core IV conditions. Models differ depending on the exact setting (e.g. continuous or binary outcome, case-control or cohort study) and target of inference (e.g. local versus population parameter, causal average effect versus causal risk ratio). Furthermore, different parameters can be targeted by the same estimator under different assumptions so attention should be paid to the modelling details (Hernán and Robins 2006a; Brookhart and Schneeweiss 2007; Angrist and Pischke 2009; Didelez et al. 2010; Clarke and Windmeijer 2012). It is hence important to be clear about what parameter is being targeted and what assumptions are being made for any particular analysis.

#### IV estimation in linear and additive structural models

Here, we give a brief overview of the simplest and most popular case, the linear additive structural model. Other models are discussed briefly in “Other IV models and estimators”. We call this type of model ‘structural’ because it is assumed to be valid not only under observation of but also under intervention in *X* as explained earlier. It assumes that

2 $$\begin{aligned} E(Y \mid X = x,U = u) & = E(Y\mid \mathrm{do}(X = x),U = u)\nonumber \\ & = \beta x + h(u), \end{aligned}$$

where the first equality is due to the structural assumption. This model posits that the causal effect within levels of the confounders *U* is linear in the exposure *X* without effect modification by *U* on the chosen scale, i.e. individuals in confounder subgroups such as men/women, drinkers/non-drinkers or older/younger, all react similarly to exposure. The unobserved confounders can predict or affect the outcome *Y* in an arbitrary way *h*(*u*). The model implies that the average causal effect for a one unit increase in *X* is identified as $\mathrm{ACE}(x,x+1)=\beta$.

The parameter $\beta$ cannot immediately be estimated from the above as we have no data on *U*. Moreover, we cannot obtain an unbiased estimate of $\beta$ from a regression of *Y* on *X* due to correlation between *U* and *X*. Here, the IV comes into play. Exploiting the core IV conditions, it follows from the above structural linear and additive model that

$$\begin{aligned} \beta = \frac{\mathrm{Cov}(Y,G)}{\mathrm{Cov}(X,G)}, \end{aligned}$$

suggesting a simple estimator because the ratio of the covariances is in fact equal to the ratio of the regression coefficients from regressions of *Y* on *G* and of *X* on *G*:

3 $$\begin{aligned} {\hat{\beta }} = \frac{{{\hat{\beta }}}_{Y\mid G}}{{{\hat{\beta }}}_{X\mid G}} . \end{aligned}$$

This result has been known for a long time (Wright 1928; Wald 1940; Wooldridge 2002), but see Didelez et al. (2010) for a proof using the same notation as above.

The so-called ratio estimator (3) is simple to compute and has desirable statistical properties in that it is consistent. However, we now see why we need core IV condition 2: if the denominator is close to zero (relative to the measurement scale) the whole expression becomes very unstable and the variance of ${\hat{\beta }}$ then tends to infinity. The denominator $({{\hat{\beta }}}_{X\mid G})$ will be close to zero if the instrument *G* does not strongly predict *X*; this is known as a weak instrument. It is plausible and can be shown formally, that the strength of the instrument (as measured by the proportion of variation in *X* that it explains) and the amount of confounding are inversely related: if *U* explains a lot of the variation in *X*, then there is not much variation left for *G* to explain (Martens et al. 2006). Moreover, use of a weak IV leads to loss of power for detecting a causal effect, if present, and also tends to bias the IV estimate of causal effect towards the naïve or ordinary least squares estimate which is precisely the bias that an IV analysis is trying to circumvent (Bound et al. 1995). For a single IV, the above ratio estimator is equivalent to the two-stage-least-squares (2SLS) estimator: predict *X* from a linear regression of *X* on *G*, and then carry out a linear regression of *Y* on the predicted values ${\hat{X}}$. The latter has the advantage of being generalisable in a straightforward way to multiple instruments, but, unlike the ratio estimator, requires joint data on *G*, *X* and *Y*.

Instrument strength is related to the (adjusted) $R^2$ from the regression of *X* on *G* and the corresponding *F*-statistic for the null hypothesis that the IV does not predict *X* at all. Strength is relative to sample size and hence the much-cited rule-of-thumb of $F \ge 10$ for an acceptably strong IV is valid for a single IV if the focus is on the actual level of an IV-based test. It does not provide a significance test of the null hypothesis at the same level for multiple IVs (Staiger and Stock 1997). The two values, $R^2$ and *F*, should always be reported in any MR analysis but it is important to note that neither constitutes a definition of a strong/weak IV. Also, any data-driven approach to modelling the regression of *X* on *G* based on optimising $R^2$ and *F* will bias the analysis (Sheehan and Didelez 2011).

#### Multiple instruments

In many applications of MR, it is possible that several variables, $G_1,\ldots , G_K$, are plausible candidates as instruments for the effect of *X* on *Y*. It is especially tempting to use databases of published GWAS results to identify numerous potential instruments for the same exposure-outcome relation.

Multiple instruments offer some potential benefits, for example with regard to the plausibility of assumptions. In particular, if each $G_k$ separately satisfies the core IV conditions, then they should all estimate the same causal effect and so separate estimates of the causal effect parameter should be roughly similar. Note that this reasoning presumes a homogenous causal effect as implied by the linear additive structural model (2). Under this model, large differences in the resulting estimated values possibly indicate that some of the core IV conditions may be violated for some of the instruments or, if they are all believed to be valid, that the model is incorrect. When the multiple IVs are independent, then this is the basic idea underlying an over-identification test (Sargan 1958; Hansen 1982). Under model (2), similar estimates of the causal effect parameter thus provide evidence against bias due to pleiotropy or linkage disequilibrium but not necessarily due to population stratification. Of course, this procedure will still fail to detect problems if the separate IV estimates are all biased in exactly the same way (Palmer et al. 2012; Glymour et al. 2012).

When it is implausible that the causal relationship between *X* and *Y* can be summarised in a single parameter, such as when it is not linear or when there is effect modification by observed covariates so that model (2) does not hold, we can exploit multiple instruments to estimate more parameters. Hence, multiple instruments can be used to estimate more complex causal models. However, in such a case all instruments have to be sufficiently strong as well as sufficiently unrelated to provide the required increase in information.

#### Multiple IVs and 2SLS

With a single IV, the 2SLS estimator is asymptotically unbiased for the average causal effect but it is subject to finite sample bias which is exacerbated when the instrument is weak (Bound et al. 1995). Under model (2) with ${\mathbf {G}}$ the vector of IVs, 2SLS estimation easily accommodates multiple IVs by fitting a regression of *X* on all $G_1,\ldots , G_K$ jointly in the first stage. The additional instruments can serve to reduce weak-IV-bias provided they also increase the amount of variation explained in the exposure *X*. However, adding very weak, or virtually ‘redundant’ IVs, could actually increase the bias as this is likely to lead to over-fitting the first-stage regression and renders the occurrence of an accidental correlation between an instrument and unobserved confounding *U* more likely. Other estimators, such as the limited information likelihood and continuous updating estimators, have been shown to be more robust to weak IV bias (Sheehan and Didelez 2011; Davies et al. 2015).

#### Multiple IVs and allele scores

In MR applications it has become popular to use genetic risk or allele scores composed of several SNPs rather than a single genetic variant. Such a score *S* is given as the weighted average of the multiple IVs/SNPs: $S=\sum _k w_k G_k$. The IV estimate of $\beta$ is then obtained by regressing *X* on *S* at the first stage and then proceeding as usual. For this procedure to result in a consistent estimator of the causal effect, the score *S* needs to satisfy the IV core conditions; in particular it must be sufficiently informative for *X* (core condition 2) as measured by the $S-X$ association. A violation of the other core conditions will typically occur, if one or more of the $G_k$’s are themselves not valid IVs, so that we can say that all $G_1,\ldots , G_K$ need to be valid for the score to be valid (Swanson 2017).

**Fig. 6:**
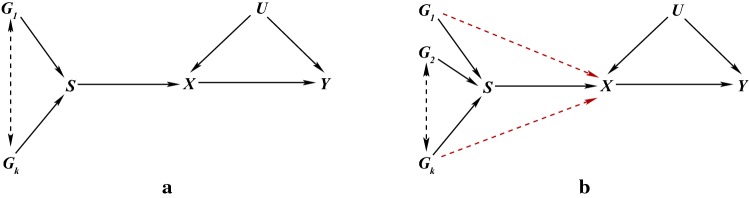
DAGs depicting an allele score *S* where **a***S* is a perfect summary of possibly correlated $G_1,\ldots ,G_k$ and **b***S* does not capture all the information in $G_1,\ldots ,G_k$. In both cases, *S* is still a valid IV for the causal effect of *X* on *Y* with unobserved confounding *U*

To see the advantage of using an allele score, first note that 2SLS is equivalent to determining the weight $w_k$ of each IV $G_k$ as the regression coefficient from a multiple regression of *X* on $G_1,\ldots , G_K$ jointly on the same data used for the whole analysis. As mentioned above, this easily gives rise to weak IV bias due to overfitting. Typically, however, the weights for an allele score are determined in a different way and several suggestions for how to do this have been proposed. If joint data are not available, as is often the case, one could obtain the weights for each SNP $G_k$ from a simple regression of *X* on $G_k$ alone. This is equivalent to 2SLS if the instruments are independent, but will not suffer from weak-IV-bias if a different data source is used for these *K* individual regressions than for the second stage. In principle, IVs do not have to be independent to be combined into a valid allele score in a one-sample setting (Fig. 6a). However, the weights for correlated IVs should ideally be obtained from a regression of *X* on $G_1,\ldots ,G_K$ jointly and based on external data (Burgess et al. 2016). More generally, one could make use of other external information, e.g. other data sources or subject matter knowledge, to determine the weights. The number of parameters could be reduced by restricting the weights to be a constant $w_k\equiv w$ for all $G_k$, as in an unweighted score, or by partitioning $G_1,\ldots , G_K$ into two groups, one with (the same) high weight and the other with low weight. Most allele scores implicitly assume an additive genetic model whereby each SNP has an approximately additive per allele effect on *X*: an unweighted score assumes similar per allele effects across all SNPs. Biological knowledge can be incorporated to distinguish between SNPs that can be regarded as ‘major genes’ and thus fitted separately in the first-stage regression and those that are polygenic and can be combined into an allele score (Pierce et al. 2010; Palmer et al. 2012). Advantages of using allele scores mainly stem from either using external data or restricting the weights and hence reducing the number of parameters, as this alleviates weak IV bias provided all SNPs in the score are themselves valid IVs (Pierce et al. 2010; Palmer et al. 2012; Burgess and Thompson 2013).

Moreover, MR analyses based on allele scores seem to be less sensitive than 2SLS analyses to misspecification of the first-stage regression, i.e. using the ‘wrong’ score, but they are very sensitive to the choice of variants for inclusion in the score and to the derivation of the weights (Burgess and Thompson 2013). A perfect score would have the property that it fully summarises the information in $G_1,\ldots ,G_K$ for predicting *X*, implying $X\bot \!\!\!\bot (G_1,\ldots ,G_K)|S$. This is unlikely to hold if restricted weights or an unweighted score are used, but the resulting loss of information often outweighs the danger of introducing bias due to overfitting an overly complex first stage model or score. It is important to note that *S* is still a valid IV even if $X\bot \!\!\!\bot (G_1,\ldots ,G_K)|S$ does not hold (see Fig. 6b) as long as the $G_1,\ldots ,G_K$ are valid IVs. It would be a problem for methods requiring a causal and unconfounded IV.

#### Multiple IVs and two samples

Up to now, we have mostly assumed a ‘one-sample’ scenario where individual-level data are available on all observable quantities, *G*, *X* and *Y*. The ratio estimator (3) can also be used in a ‘two-sample’ setting where summary data on the *G*–*X* and *G*–*Y* associations are taken from different studies under the assumption that the two underlying study populations are broadly similar (Hartwig et al. 2016). This lends itself to exploitation of potentially very large numbers of publicly available genome wide association studies (GWAS) providing summary information on associations between candidate IVs $G_k$ and exposure *X* and outcome *Y* of interest. For example, in a recent MR study of the effect of age at puberty on asthma risk (Minelli et al. 2018), potential instruments were initially selected from a large published genome wide meta-analysis and supplemented through a literature search for additional relevant genetic studies using curated collections such as the NHGRI GWAS Catalog (Welter et al. 2014) and HuGE Navigator (Yu et al. 2008). The MR-Base platform (http://www.mrbase.org) has been specifically developed for MR analyses and has over 11 billion SNP–trait associations from almost 2000 GWAS to choose from (Hemani et al. 2018). In this situation, MR with multiple independent IVs can be viewed as a meta-analysis where the individual ratio estimates corresponding to each $G_k$ can be combined into a pooled inverse variance weighted (IVW) estimate (Burgess et al. 2017a; Thompson et al. 2016, 2017). The one-sample over-identification test can be replaced by a standard $\chi ^2$ test for heterogeneity such as $I^2$ or Cochran’s *Q*-statistic used in meta-analysis (Del Greco M et al. 2015; Bowden et al. 2016b, 2017). Summary data methods can also be extended to include correlated SNPs and to construct allele scores (Burgess et al. 2016; Zhu et al. 2018).

#### Allowing for invalid IVs

The more SNPS that are considered as IVs in an MR analysis, the more likely it is that they will not all satisfy the core IV conditions. In the one-sample setting, the method of Kang et al. (2016) (and further developed in Windmeijer et al. (2018)) permits identification of the causal effect as long as fewer than 50% of the IVs are ‘invalid’ without the need to identify the offending IVs. The approach essentially penalises SNPs with suspected pleiotropic effects and downweights them in the analysis. Analogous robust approaches for the two-sample setting include: MR-Egger regression (Bowden et al. 2015) which can potentially cope with 100% invalid IVs under a strong assumption about the suspected pleiotropic effects; a weighted median approach (Bowden et al. 2016a) again assuming less than 50% invalid IVs; and mode-based estimation (Hartwig et al. 2017) which is consistent when the largest number of ‘similar’ individual SNP-based ratio estimates derive from valid IVs. All these robust approaches yield estimates that are less precise than 2SLS or IVW estimates, but should be carried out as part of a sensitivity analysis to support or question causal conclusions (Burgess et al. 2017a). They all make different and strong assumptions so we would go one step further and suggest that more weight should perhaps be given to analyses that do not rely so heavily on parametric assumptions (Clarke and Windmeijer 2012).

Because of the increasing availability of multiple candidate genetic IVs, development of methods for incorporating multiple IVs—particularly in the two-sample setting—have been mainly restricted to the MR literature. Attention is now turning to applying such approaches to the one-sample setting as intensive phenotyping of genetic association study populations is taking place and individual level data on instrument(s), exposure and outcome can reasonably be expected in many situations. It should be noted that establishing the validity of a set of IVs requires additional care and commonly used terms such as ‘all valid’ and ‘some invalid’ are neither used consistently nor explicitly defined.

### Other IV models and estimators

The linear and additive structural model of “IV estimation in linear and additive structural models‘’ may often be plausible, at least as an approximation, for a limited range of *X* values. It can be shown that 2SLS has very good robustness properties under this model even when certain aspects, such as the first stage model or the way in which measured covariates enter the model, are misspecified (Vansteelandt and Didelez 2018).

These desirable properties of 2SLS do not typically carry over to non-linear models which are used, for instance, when the outcome *Y* is binary. For binary outcomes, a linear approach would still estimate the ACE or causal risk difference, but we may then prefer to report the CRR or COR, requiring non-linear models. Under certain parametric assumptions about the exposure distribution and using a log-linear model for the second stage regression, the CRR can be targeted by a two-stage regression or ‘ratio-type’ estimator (Didelez et al. 2010). The main problem for the non-linear case is that the relationship between the two regressions (*Y* on *G*, and *X* on *G*) and the relevant causal parameter, CRR or COR, is no longer straightforward and estimators derived from these two regressions are typically biased (Vansteelandt and Goetghebeur 2003; Martens et al. 2006; Palmer et al. 2011b; Vansteelandt et al. 2011; Harbord et al. 2013). This is also true when the focus is on a local, or ‘complier’ odds ratio (Cai et al. 2011). There are other IV methods dealing with binary outcomes, or more generally non-linear structural models, but they are less intuitive than the ratio estimator, and less simple to construct. The CRR, for example, can also be estimated under the weaker assumptions of a structural mean model or using a generalised method of moments estimator but identification problems can arise as the estimating equations sometimes have multiple solutions (Hernán and Robins 2006a; Clarke and Windmeijer 2010, 2012; Burgess et al. 2014).

Targeting the COR poses additional problems due to the non-collapsibility of odds ratios and the situation becomes even more complicated if data on (*X*, *Y*, *G*) are obtained from a case–control study where bias can be induced through conditioning on the outcome *Y*. In a case–control setting, the distribution of confounders in the control group is typically different from that in the general population due to over-recruitment of cases and this can induce an undesired association between the IV *G* and the unmeasured confounders *U* (Didelez and Sheehan 2007b). Here, ORs have to be used despite the problems induced by selecting on case status since other measures of association are even more sensitive to retrospective sampling (Burgess et al. 2017b). When good estimates of disease prevalence or population allele frequencies are available, an MR analysis can be re-weighted to yield reliable estimates of the COR (Bowden and Vansteelandt 2011). Recent advances have been made using IVs for survival outcomes. Non-collapsibility of the hazard ratio in the popular Cox model is problematic and requires an approximate approach (Martinussen et al. 2019) whereas additive hazard models behave more like 2SLS (Tchetgen Tchetgen et al. 2015; Martinussen et al. 2017). They all require individual-level (one sample) data and are restricted to a single IV.

Bayesian approaches to MR analyses have also been proposed (Burgess et al. 2010; Burgess and Thompson 2012; Jones et al. 2012) and recent work addresses the issue of dependent IVs (Shapland et al. 2019) and invalid IVs with pleiotropic effects (Berzuini et al. 2019). These methods have yet to gain popularity in applied studies, possibly due to the unavailability of user-friendly software but also, perhaps, because these approaches are fully parametric requiring a complete specification of the likelihood (which implicitly or explicitly includes the unobserved confounding) together with prior distributions on all parameters in the model. Inferences are hence very sensitive to the modelling assumptions and prior information.

## Discussion

It has never been easier or more tempting to conduct a Mendelian randomisation study. Recent developments in genetic epidemiology have yielded billions of SNP–trait associations that can be trawled to produce hundreds of potential IVs for MR studies. Two-sample analyses are increasingly easy to conduct as statistical packages are being made more widely available. Indeed, the MR-Base platform integrates a database of GWAS results with an interface that permits automated MR analyses using several of the methods mentioned in “Multiple instruments”. Although the authors explicitly warn against this, there is a danger that MR will become a ‘black box’ analysis (Hemani et al. 2018). Furthermore, the more IVs that are included, the more problems that can potentially arise and hence the more important it is to be clear about the research questions of interest, the causal parameter being targeted and the modelling assumptions that underlie any causal conclusions (Swanson et al. 2017). To this end, it could be helpful to think about what randomised trial would be conducted to investigate these questions were such a trial possible (Hernán and Robins 2016). We have commented on the differences between MR and randomised trials in this paper. However, both should give careful consideration to the target population, the intervention under consideration and the causal effect of interest. Also, many of the issues to do with reporting MR analyses are similar to those for trials and have been commented upon by many authors (Swanson and Hernán 2013; Glymour et al. 2012; Davies et al. 2013; VanderWeele et al. 2014).

Causal inference always relies on special assumptions. Practitioners tend to dislike the fact that some of these are not verifiable empirically. However, the more familiar assumption of ‘there is no unmeasured confounding’ underlying a standardisation or propensity score analysis, for example, is just as untestable as the IV core conditions. The limitations of such assumptions need to be fully understood (Hernán and Robins 2006a) and sensitivity analyses—to whatever untestable assumptions have been made—should be routinely conducted (Lash et al. 2009; Silva and Evans 2016). In particular, justification for carrying out an MR analysis in the first place should always be provided as an IV analysis can be more biased than a naïve analysis if there is little or no unmeasured confounding (Brookhart et al. 2010). The IV core conditions should be routinely evaluated in a systematic way (von Hinke et al. 2016) and care should be taken when establishing validity of sets of SNPs jointly for use in an allele score, for example. As is standard practice in observational epidemiology, evidence should be obtained from as many different sources as possible, assumptions should be clearly discussed and reasons for accepting (or refuting) them provided (Glymour et al. 2012).

We have also argued that the structural assumption required to link the causal and observational regimes is seldom mentioned, even though it is regularly assumed, and it does have implications for the type of intervention that can be considered. For the effect of age at puberty on asthma, for example, a proposed pharmaceutical intervention might invalidate this assumption and the desired causal effect on asthma may not be achieved. Violation of the first and third core conditions so that $G \bot \!\!\!\bot \!\!\!\!/\;Y \mid (X,U)$ is often modelled as a simple direct effect of *G* on *Y* in sensitivity analyses with a single parameter representing the association. While this makes sense mathematically, the violation can also occur via *U* (see Fig. 1a) and, as we have seen in “Establishing validity for a candidate IV”, can arise for different biological reasons. What one is willing to assume about the size and direction of such effects for these sensitivity analyses should be informed by what is biologically most plausible.

Due to the fact that many published epidemiological findings cannot be replicated, there is a danger that the current focus on replication may get confused with actual verification. It has hence been suggested that a range of different approaches—including MR—should always be used to verify epidemiological results in a process called ‘triangulation’ (Lawlor et al. 2016). We would agree with this but would stress that it is only useful if the assumptions of the different approaches involved are clearly justified.

Mendelian randomisation has enormous potential for causal inference in observational epidemiology but it should never be an automated process based on downloaded SNP–exposure and SNP–outcome associations. The underlying assumptions of any analysis should be systematically inspected for every single study and biological knowledge, in particular, should be incorporated. Indeed, the selection of SNPs to include as IVs and the assessment of their (possibly joint) validity are probably more important issues than the particular choice of analysis method (Burgess et al. 2017b). A test for the null hypothesis of ‘no causal effect’ should always be carried out and bounds for the causal effect should be calculated whenever possible. This is because they both inform on what can be inferred from core IV conditions and the data alone without making any additional (semi-) parametric assumptions. Point estimates require further—and typically—strong assumptions. More importantly, results that depend solely on specific and unverifiable parametric assumptions will not necessarily be replicable.

There is a tendency in the MR literature to shy away from drawing causal conclusions, even when these are statistically supported, and referees often advocate caution in interpreting results causally. We agree with Swanson and Hernán (2018) that the whole point of an MR analysis is to draw causal conclusions. Otherwise, why not base cautious conclusions on a standard regression analysis which is a lot simpler? Indeed, it is essential to be able to use the word ‘causal’ for rigorous and meaningful epidemiological research (Hernán 2018). MR estimates of causal effects should always be interpreted causally, but it should be made clear that they are conditional on the particular assumptions underlying the analysis. If researchers are not happy to make such assumptions they should not produce a point estimate (VanderWeele et al. 2014) and should base their inferences on the test of the causal null, the bounds and on sensitivity analyses.
